# Risk Factors for Dental Caries Experience in Children and Adolescents with Cerebral Palsy—A Scoping Review

**DOI:** 10.3390/ijerph19138024

**Published:** 2022-06-30

**Authors:** Sarah Cui, Rahena Akhter, Daniel Yao, Xin-Yun Peng, Mary-Anne Feghali, Winnie Chen, Emily Blackburn, Elizabeth Fieldja Martin, Gulam Khandaker

**Affiliations:** 1 Sydney Dental School, Faculty of Medicine and Health, The University of Sydney, Camperdown, Sydney, NSW 2006, Australia; scui5507@uni.sydney.edu.au (S.C.); dyao2737@uni.sydney.edu.au (D.Y.); xpen0588@uni.sydney.edu.au (X.-Y.P.); maryanne.feghali@health.nsw.gov.au (M.-A.F.); winnie.chen2@health.nsw.gov.au (W.C.); ebla7377@uni.sydney.edu.au (E.B.); elizabeth.martin@sydney.edu.au (E.F.M.); 2 Central Queensland Public Health Unit (Rockhampton), Rural and District Wide Service, Central Queensland Hospital and Health Service, Rockhampton, QLD 4700, Australia; gulam.khandaker@health.qld.gov.au

**Keywords:** dental caries, cerebral palsy, risk factors, children and adolescents, review

## Abstract

Cerebral palsy is a developmental motor disorder which has far-reaching impacts on oral health. This scoping review examined the extent of research undertaken regarding the risk factors affecting dental caries experience in children and adolescents with cerebral palsy. Data were obtained from the electronic databases Web of Science and PubMed, using 10 search strings, for studies published between 1983 and 2018. Eligible studies were required to have investigated caries in children under 18 with cerebral palsy, as well as be written in English. 30 papers published were identified for inclusion in the review. These included 23 cross-sectional, 6 case–control, and 1 longitudinal study. Studies were categorized into six domains of risk factors: socioeconomic status (SE); cerebral palsy subtype (CPS); demographics (D); condition of oral cavity (OC); dental habits (DH); nutrition and diet (ND). This review was conducted and reported in accordance with Preferred Reporting Items for Systematic reviews and Meta-Analyses Extension for Scoping Reviews (PRISMA-ScR) guidelines. The most significant risk factors were caregiver-related education levels, oral health literacy, and sugar intake; this underlines the important role of special education and dental awareness in reducing dental caries incidence in CP children. Other factors showed divergent findings, highlighting the need for standardization and culturally specific studies in future literature.

## 1. Introduction

Cerebral palsy (CP) is a permanent, developmental disorder which arises from brain damage during infancy [1]. Children with CP are particularly vulnerable and often exhibit poor oral health and systemic health consequences as a direct result of their disability [2]. Based on the location of motor deficit, CP is classified into quadriplegia, diplegia, and hemiplegia. It is further classified into spastic, dyskinetic, ataxic and mixed types, dictated by neurological damage [2,3,4]. These difficulties remain throughout the patient’s life, presenting problems with general self-care and oral health maintenance.

Caries is a multifactorial disease involving past and current caries experience, diet, fluoride exposure, presence of cariogenic bacteria, salivary status, and sociodemographic influences [5]. Other factors include food consistency, high-sugar beverage consumption, long-term oral medications with xerostomic potential, oromotor dysfunction, and difficulty maintaining daily oral hygiene [6]. 

The existing literature suggests that CP children generally have poorer overall dental health due to caries than non-CP children, with more extractions, poorer quality restorations of decayed teeth and worse oral hygiene [7,8]. This increased incidence of caries may be due to neuromuscular problems, changes in structure to the orofacial region, feeding problems, difficulties with maintaining oral hygiene, and barriers to oral care access [1,9]. Carvalho et al. [10] did not find any significant association between the CP type and toothbrushing frequency, as oral hygiene was carried out by caregivers in 73.1% of cases due to reduced manual dexterity, which corroborates a study by Camargo and Antunes [11,12]. Santos and Nogueira [13] found that quadriplegic individuals showed a mean decayed–missing–filled (DMF) score that was twice that of the hemiplegic group (5.8 versus 2.5), suggesting that the severity of neurological damage relates to greater risk of oral disease.

Reduced access to appropriate dental care and the ability to maintain personal oral hygiene necessitates focused research into promoting oral health [7]. The Oral Health-Related Quality of Life of children with CP is negatively impacted by the severity of dental caries, communication ability, and low family income [9]. Although higher caries incidence has been revealed in this population [14], a paucity of studies fully incorporated the array of risk factors that can affect subpopulations of CP sufferers. These risk factors were identified as socioeconomic status, CP subtype, demographics, condition of oral cavity, dental habits, and nutrition and diet [6,10,11,13,14,15].

Understanding the risk factors affecting caries prevalence in children and adolescents with CP forms the groundwork of providing quality oral health education. The present scoping review aimed to summarize previous findings to better understand the significant risk factors that contribute to the high incidence of dental caries in this vulnerable population. Scoping reviews examine the range and nature of existing evidence, identify gaps in knowledge, and aid in planning future systematic reviews. In this way, the current study synthesizes existing risk factors into clear domains from which further investigation can be made.

## 2. Materials and Methods

### 2.1. Data Collection

This review followed the PRISMA-ScR (Preferred Reporting Items for Systematic reviews and Meta-Analyses extension for Scoping Reviews) protocol, in light of deploying a transparent and systematic means of assessing chosen papers within the review (Supplementary Figure S1). PRISMA comprises a four-phase flow analysis marked against a checklist of components indicative of a robust methodology, with an extension for scoping reviews applied to the current study. Electronic searches were conducted using search engines Web of Science (WoS) and PubMed (Supplementary Figure S2).

**Figure 1:**
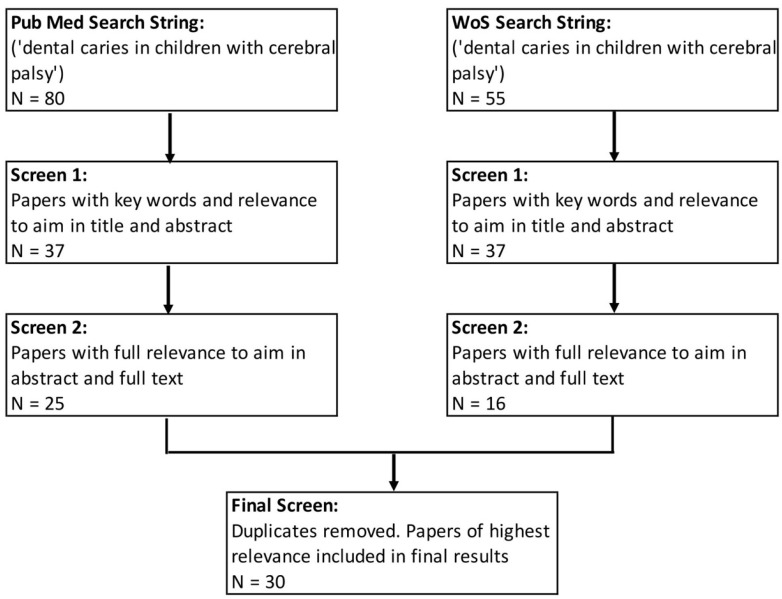
Details of the screening process employed using search engines, Web of Science (WoS) and PubMed and 10 different key words are shown. With each successive step, a screen was performed, narrowing the selection of papers based on highest relevance.

In the first screening process, abstracts and titles were scanned for keywords such as ‘dental caries’, ‘cerebral palsy’, ‘oral motor function’, ‘demographics’, ‘cerebral palsy sub-type’, ‘nutrition and diet’, ‘socioeconomic’, and ‘dental habits’. The analysis of full texts was performed in the second screening. Duplicate papers present in both PubMed and WoS were accounted for. The search was restricted from 1983 to 2018 to provide a 35-year time frame for analysis. Six reviewers independently reviewed each article with calibrated Microsoft Excel forms to chart data, identify risk factors, and note study characteristics. Findings were then presented in group workshops to reach a consensus on study selection and categorization of risk factors (Figure 1). This included analyzing demographics, independent and dependent variables, significant findings, and the conclusions of each paper. Risk of bias for each article was similarly conducted based on the National Heart, Lung, and Blood Institute (NHLBI) Quality Assessment Tools for Cross-Sectional Studies and Case–Control Studies, and results were calibrated in group discussion. Journals that were poorly translated or not available in English were excluded due to the potential for misinterpretation by the reviewers.

### 2.2. Data Analysis

After evaluation of these parameters, reviewers identified key themes and risk indicators for dental caries. The key findings were categorized under six broader domains: socioeconomic status, CP subtype, demographics, condition of the oral cavity, dental habits, and nutrition and diet. Socioeconomic status included variables such as domestic income, education levels and household crowding. All factors regarding classification of CP grade and severity were categorized under CP subtype. Demographics pertained to individual features of age and sex. Oral cavity condition included the level of oral motor control, including biting reflexes and drooling, as well as salivary markers. The frequency of toothbrushing and dental visits were categorized under the dental habits risk factor. Finally, nutrition and diet included variables regarding sugar intake and diet consistency.

### 2.3. Inclusion Criteria

- Participant characteristics: individuals 18 years or under with CP
- Study characteristics/contents: research papers investigating potential factors influencing caries prevalence
- Studies written in English or with an available English translation

## 3. Results

The study selection process yielded 30 papers of the highest relevance (Figure 1). These included 23 cross-sectional, 6 case-control, and 1 longitudinal study published between 1983 and 2018. Most studies focused on two or more risk factors associated with dental caries in children and adolescents with CP (Table 1). 

### 3.1. Socioeconomic Factors (SE)

There were varied findings with regards to the effects of domestic income on the prevalence of dental caries in CP children and adolescents. Several studies reported that low socioeconomic backgrounds have no significant effect on dental caries in children and adolescents with CP [15,16,17]. In particular, two studies found that caregivers’ monthly income was not significantly associated with dental caries prevalence [11,18]. However, a recent study in Brazil reported a significant relationship between familial income of less than R$1500 and dental caries, but had higher risk of bias due to clinical data being obtained from records only [19]. A proportion of 78.3% of families in another study had a domestic income three times less than the Brazilian minimum wage [11].

Most studies concluded that caregiver education levels shape attitudes and inform oral health practices, ultimately impacting dental caries prevalence in CP children and adolescents [11,18,20,21,40]. Three studies found that low education levels of caregivers (<8 years) significantly increased caries prevalence in CP children [11,18,19]. One study also observed that untreated dental caries increased with having siblings and living in crowded households [21]. Another article reported that although 54.3% of mothers had completed primary education, most were unaware of the importance of oral hygiene, as reflected in the poor dental habits of their children and adolescents [20].

Overall, low caregiver education levels appear to significantly increase risk of caries in CP children (Table 2). Thus, the most significant socioeconomic risk factor is parental educational levels, with the preventable nature of caries suggesting the need for an approach that is centered around increasing oral health awareness and training caregivers.

### 3.2. Cerebral Palsy Subtype (CPS)

Several studies investigating caries incidence between quadriplegic, diplegic, and hemiplegic patients with spastic CP discovered no significant difference [10,11,22,23]. However, in one longitudinal study [11] validity may have been compromised by a high subject dropout rate. One study also found that hemiplegia, which presented without the biting reflex, had significantly lower caries than the other physical subtypes of CP [13]. Akhter et al. also found that children and adolescents with spastic quadriplegia showed higher caries experience when compared to other spastic subtypes [24]. Further targeted studies of neurological CP classification effects on caries experience in children and adolescents will clarify this association.

Spastic-type CP was found to be the most common CP type in current study with patients presenting with muscle spasticity showing higher caries experience [18,25]. Two recent studies have investigated caries risk within the Gross Motor Function Classification System (GMFCS) for CP, finding that children in levels IV/V presenting high motor dysfunction had significantly greater risk of caries and poor oral hygiene when compared to control groups [24,26].

Furthermore, studies targeting the correlation between intellectual disability and caries prevalence, using the Raven Colored Progressive Matrices Test (RCPMT), found a positive relationship [15,16]. The same studies found a low association between motor ability and dental caries experience. A Taiwanese study similarly found that intellectual disability had a significant effect on caries, and interestingly, moderate CP sufferers required the highest treatment needs when compared to severe CP sufferers across age groups [27]. In contrast, one study [28] found that both lower general motor ability and oral motor performance presented higher caries experience in spastic CP children, and another suggested that physical disability presents higher caries risk than intellectual disability [29].

### 3.3. Demographics (D)

Several studies showed that higher age has a positive correlation with the incidence of dental caries, especially when examining young toddler groups through infancy [23,27,30,31]. Recent research has also shown that the proportion of affected CP children and adolescents doubled upon progression from 2–6 years to 7–11 years, highlighting the importance of prevention at an early age [24]. This early onset of caries often coincided with other oral health diseases such as gingivitis that were more prevalent at a later age [26].

However, one study found the contrary, proposing that there is no significant association between age and caries prevalence [28]. Another study found that the younger children in the 4–19-year-old group had significantly higher DMF values when compared to the older children and adolescents [6]. Sex was not identified as a risk factor in most studies that were reviewed [23,27,32]. Despite this, it was found in China [17] that female CP children and adolescents were 1.9 times more likely to have dental caries.

### 3.4. Condition of the Oral Cavity (OC)

A positive correlation between insufficient oral cavity function and caries incidence has been highlighted. In a study on Brazilian children and adolescents with CP, higher DMF/dmf values were associated with greater oral motor function impairment, which may influence oral clearance times, and subsequently caries prevalence [6]. A direct association between caries prevalence and substandard oral motor performance (including poor mastication, swallowing, and mouth closure) was also reported [28]. A more severe biting reflex may also exacerbate oral hygiene maintenance and may heighten the risk of oral disease in CP children [13]. Conversely, one study identified a negative correlation between compromised oral cavity conditions and caries incidence, where bruxism and dental attrition were found to be associated with a lowered risk of caries [33].

The physiochemical properties of saliva may also impact caries prevalence, with CP children and adolescents exhibiting elevated levels of salivary sialic acid, lower total antioxidant levels, and significantly poorer oral health [34]. Total antioxidant capacity was propounded to be inversely related to dental caries incidence in CP children [35]. Salivary flow rate was also found to be negatively associated with caries prevalence, while saliva osmolality may be a potential risk indicator for caries development in CP children and adolescents [28]. Anomalies in salivary quantity was found to have a strong, negative correlation with Dmft/DMFT values for CP children, and salivary pH was found to negatively correspond with caries occurrence in the primary dentition [35]. A positive correlation between the drooling of saliva and dmft has also been reported [26]. Interestingly, botulinum toxin A, a medication used to treat sialorrhea, was found to lower salivary pH and increase caries prevalence in neurologically impaired children, but not specifically CP, and was therefore not included in the final selection [41].

While drooling in CP children and adolescents was elevated and potentially predisposed them to poorer oral hygiene, it did not heighten caries occurrence in a study conducted on CP children aged five to eighteen [36]. Additionally, despite CP individuals presenting with elevated salivary osmolality, they did not exhibit elevated caries [37]. Overall, while most studies promoted a correlation between caries prevalence and conditions/factors of the oral cavity, some studies presented divergent findings.

### 3.5. Dental Habits (DH)

Inadequate toothbrushing frequency was found to be a significant risk factor affecting caries experience in CP children in most studies [17,24]. This has been attributed to compromised orofacial motor dysfunction and decreased intraoral sensitivity, which can make it harder to uphold daily oral hygiene [18,24,26]. This is consistent with findings that CP children with more severe functional motor impairment were at higher risk, with more caries prevalent in the lower posterior teeth, which are often the hardest to brush [24,26]. Interestingly, other studies found that the level of functional motor impairment did not correlate with the toothbrushing frequency or oral health of CP children, which can be explained by the dependency of most CP children on their caregivers for brushing [10,14,20]. Further, some studies refuted that the frequency or difficulty of brushing was related to caries experience [18]. Instead, CP children who experienced communication problems with their caregivers were at highest risk, suggesting that difficulty in explaining their oral conditions might explain the higher caries prevalence; however, the authors noted that this hypothesis remains to be tested [18].

Caregivers’ educational levels and perception of oral health were found to be correlated with increased caries experience, likely because oral health literacy in caregivers is important in upholding good dental habits [18,20]. This is supported by findings that show that brushing frequency and duration of dental visits in CP children reflected those of their mothers’ [20]. However, the current evidence linking the duration and frequency of dental visits to caries experience in CP children remains mixed, with most studies finding no correlation [17,18,24].

### 3.6. Nutrition and Diet (ND)

Studies have shown mixed results regarding the association between sugar intake and caries incidence. A Norwegian study of preschool children and adolescents with disabilities, including CP, reported that caries prevalence correlated with greater carbohydrate intake but had no association with the amount of sweetened medication [38]. Furthermore, in cross-sectional studies of Brazilian institutionalized CP children and adolescents, a higher sugar consumption correlated with increased caries incidence [11,21]. However, another Brazilian cross-sectional study showed an insubstantial correlation but obtained their data from dental records only [30]. These mixed results may be explained by the different age ranges recruited in the studies [11,21,30]. Those showing a positive correlation between sugar intake and caries recruited 2–17 year-olds [11,21], whilst the study finding no correlation investigated 1–5 year-old CP patients [30].

Similarly, views regarding the association between diet consistency and caries prevalence diverge. A bivariate analysis between diet consistency (solid or liquid) of institutionalized CP children and adolescents and caries presence revealed no significant correlation [11,18,39]. However, a study with non-institutionalized Brazilian CP children and adolescents reported that liquid diets had a significant association with dental caries, due to their increased sugar content [6].

In regard to snacking frequency, there is a general agreement amongst studies that report its lack of association with caries incidence in CP children and adolescents [17,18]. Ingesting sugary snacks before sleeping also had no relationship to caries incidence [17]. However, the lack of association may be attributed to cases where adequate dental care routine is implemented afterwards, which is consistent with the potential association between dental care habits and caries experience [17].

## 4. Discussion

This study aimed to synthesize the existing evidence about dental caries in CP children into clear domains, determine significant risk factors, and identify gaps in knowledge. Caregiver education and training, communication, sugar intake, and intellectual disability severity in the child were the main factors affecting caries experience. Snacking frequency was not found to have a significant effect on caries experience in CP children, whilst the neurological subtype of CP (spastic, dyskinetic, and ataxic) lacked substantial literature. All other risk factors presented conflicting results that require further research.

CP patients are dependent on their caregivers and their dental caries experience may be largely influenced by socioeconomic circumstances, determined by factors such as their caregiver’s education and domestic income. Although several studies found no association with dental caries, it should be noted that children with CP are disproportionately represented in lower socioeconomic groups [11,15,16,17,18]. Additionally, a study noted that dental caries impose a significant burden on the quality of life of both CP patients and their family, a burden which decreases with increasing domestic income [42].

Most studies concluded that caregiver education levels shape attitudes to dental health, ultimately impacting dental caries prevalence [11,18,19,20,21]. However, it can be argued that education levels in different countries are not equivalent. For example, while primary education may be considered sufficient in developing countries, it may be considered a low level of education in developed nations. From this, it is advisable for dental professionals to promote personalized oral health care and to support government interventions or subsidies for families affected by CP. The preventable nature of educational-related risk factors should promote an approach that is centered around improved training and awareness.

Spastic-type CP groups have increased muscle spasticity, predicting higher caries prevalence [43,44]. This may be due to involuntary muscle movement obstructing oral hygiene and treatment [18,24,25]. In support, it was also found that spastic CP children have limited and acidic salivary flow, increasing risk of caries and oral disease [45]. Additionally, earlier research found greater dental caries in mentally retarded children and adolescents, followed by CP groups and other physical disabilities [46]. A strong correlation between intellectual disability and caries found in multiple studies may be linked to a lower cooperativity and a reduced ability to understand complex instructions [15,16,27]. One study found that severe sufferers relied more on caregivers and therefore had lower caries incidence [27]. This reveals a need to target caries prevalence within different CP severities, in addition to the simplification of oral hygiene instruction. The importance of brushing in caries prevention underpins the need to improve caretaker training, communication, and oral health literacy. The use of tailored devices such as mouth props and toothbrushes with large handles may the improve ease of brushing, and therefore prevent the onset of caries.

Literature diverged on the association of dental caries with age and sex. Despite sex not being identified as a risk factor in most studies [22,27,32], it was found that female children with CP in China [17] were 1.9 times more likely to have dental caries, possibly linked to a social bias towards male children. This necessitates culturally specific evaluations of the oral health of CP children to identify vulnerable subpopulations.

Regular oral cavity function is imperative for maintaining dental hygiene and overall oral health [10,13,18]. CP children showed severe dysphagia [47,48], anomalies in swallowing [48], reduced salivary flow rate and pH [45], lingual dysfunction, prolonged and exaggerated bite reflexes, inadequate chewing [37,48], defective lip and cheek function [10], and malocclusion [49]. Such oral defects prolong the time between food intake and swallowing, compromising mastication and oral hygiene [10,13,48,50]. While there is a potential association between oral motor dysfunction and caries experience, evidence supporting a correlation with salivary characteristics is ambiguous with further research needed.

Literature has indicated a general correlation between caries prevalence and increased sugar intake and liquid diets. However, the vast majority have reported no significant difference between snacking frequency and dental caries in CP children. It should be noted that diverging results may be due to patient recruitment from specialized healthcare [11] and rehabilitation units [18], where health professionals regulate sugar intake in non-solid diets, compared to family caretakers who may not be as nutritionally aware [6]. A questionnaire-based study suggested that older CP children tended to ingest more high sugar snacks than younger children, highlighting a need for consistent sampling for age groups when investigating caries incidence and sugar intake [51]. The same study reported that caretakers often underestimated sugar levels and fermentable carbohydrates in foods such as juice boxes and biscuits, suggesting need for better caregiver education and improved food labelling [51].

Diverse cultures and locations inherently raise issues regarding the standardization of caries assessment and the classification of CP. Most studies used the World Health Organization criteria, but some also used the International Caries Detection and Assessment System with the purpose of detecting earlier stages of caries [52]. Regarding CP classification and severity, the literature was less standardized, with many studies not applying a global system at all. As such, we recommend the application of the GMFCS in future studies involving CP classification.

## 5. Limitations and Directions for Future Research

The research designs in the primary studies and this review limit the conclusions that can be drawn. Importantly, most studies examined children and adolescents from rehabilitation facilities, which do not represent a proper cross-section of the CP community. Most papers only investigated the correlation between risk factors and dental caries, and so future research targeting the direct cause and effect of these factors is required. This review only included studies published in English and was not systematic, which may have introduced bias when excluding papers based on poor translation.

## 6. Conclusions

The most prevalent factors affecting caries experience in CP individuals are linked to caregiver education and training, communication capability, sugar intake, and intellectual disability in the CP child. There was little to no evidence suggesting that snacking frequency had significant effect on caries experience, whilst the neurological subtype of CP (spastic, dyskinetic and ataxic) lacked substantial literature. All other factors examined in this review yielded conflicting results. Further investigation of the effects of different neurological subtypes of CP is needed to address gaps in knowledge. Further research should also be conducted under more specific cultural and contextual circumstances to clarify conflicting evidence.

Based on these findings, it seems imperative for caregivers of CP children and adolescents to be adequately educated on the importance of oral hygiene and prevention measures. We recommend that policy makers and special care institutions improve existing education programs for caregivers to include oral hygiene instruction using tools better suited for CP children, with an aim to reduce communication barriers and caries risk.

## Figures and Tables

**Table 1:** Relationship between risk factors and aspects correlated with caries experience (N = 30): Socioeconomic factors (SE), Cerebral Palsy Subtype (CPS), Demographics (D), Condition of Oral Cavity (OC), Dental Habits (DH) and Nutrition and Diet (ND).

Reference	Risk Factors	Aspects Significantly Correlated with Caries Experience	Aspects Not Significantly Correlated with Caries Experience	Country	Population Characteristics	Study Type	Risk of Bias
Santos et al. 2009 [6]	ND, OC, D	Low oral motor control Liquid diet Sugar intake Age	-	Brazil	108 children 4–19 y in rehabilitation	Cross-sectional	Fair
De Carvalho et al. 2011 [10]	CPS, DH	Dental health habits of caregiver	Subtype of spastic CP	Brazil	52 children 7–18 y in rehabilitation	Cross-sectional	Good
De Camargo & Antunes 2008 [11]	SE, CPS, ND	Low caregiver education Crowded household Sugar intake	Domestic income Subtype of spastic CP Diet consistency	Brazil	200 institutionalised children 2–17 y	Cross-sectional	Fair—high subject dropout rate noted
Santos & Nogueira 2005 [13]	CPS, OC	Subtype of spastic CP (hemiplegic children and adolescents with CP had less caries) Biting reflex	-	Brazil	124 non-institutionalised children 3–17 y	Cross-sectional	Fair
Sinha et al. 2015 [14]	DH	Low tooth brushing frequency Dental visit frequency	-	India	100 children, 50 with CP and 50 non-CP	Case control	Fair
Moreira et al. 2012 [15]	SE, CPS	Low intellectual ability	Domestic income	Brazil	165 children from rehabilitation centre, special school and public school	Cross-sectional	Good
Dourado et al. 2013 [16]	SE, CPS	Low intellectual ability	Domestic income	Brazil	76 CP children from rehabilitation centre compared to 89 without impairment	Case control	Good
Liu et al. 2014 [17]	SE, D, DH, ND	Sex Low tooth brushing frequency	Domestic income Snacking frequency	China	477 children from special education schools 12–17y	Cross-sectional	Fair
Cardoso et al. 2014 [18]	SE, CPS, DH, ND	Low caregiver education Neurological CP classification (spastic)	Domestic income Tooth brushing frequency Dental visit frequency Diet consistency Snacking frequency	Brazil	97 children 2–18 y from reference centre for CP children	Cross-sectional	Good
Hartwig et al. 2016 [19]	SE	Low domestic income Low caregiver education	-	Brazil	Records from university dental clinic, 7 mth–12 y	Cross-sectional	Poor – data obtained from dental records
Subasi et al. 2007 [20]	SE, DH	Low caregiver education Dental habits of mother Dental visits	-	Turkey	35 children 3-12 y	Cross-sectional	Fair
De Camargo et al. 2011 [21]	SE, ND	Sugar intake >1 sibling Low caregiver education	-	Brazil	200 children 2–17 y from non-government organisation	Longitudinal	Good
Chu & Lo 2010 [22]	CPS	-	Subtype of spastic CP	Hong Kong	65 children from special schools	Cross-sectional	Fair
Diniz et al. 2015 [23]	CPS, D	Age	Subtype of spastic CP Sex	Brazil	181 non-institutionalised children in oral health program, 4–12 y	Cross-sectional	Fair
Akhter et al. 2017 [24]	CPS, D, DH	Subtype of spastic CP (quadriplegia) High motor dysfunction (GMFCS IV-V) Age Low tooth brushing frequency	-	Bangladesh	90 children from CP register, 2–17 y	Cross-sectional	Fair
Bourke & Jago 1983 [25]	CPS	Neurological CP classification (spastic)	-	Australia	100 parents with CP attending centre	Cross-sectional	Fair
Sedky 2018 [26]	CPS, D, OC, DH	Subtype of spastic CP (quadriplegia) High motor dysfunction (GMFCS IV-V) Age Drooling Low tooth brushing frequency	-	Egypt	62 children 3–12 y in hospital	Cross-sectional	Good
Huang et al. 2010 [27]	CPS, D	Low intellectual ability Age	Sex	Taiwan	345 institutionalised children under 18 y	Cross-sectional	Good
Santos et al. 2014 [28]	CPS, D, OC	Low general motor ability Low oral motor control Low salivary flow rate High salivary osmolality	Age	Brazil	65 children 6–13 y spastic CP in rehabilitation centre	Cross-sectional	Fair
Dwizak et al. 2017 [29]	CPS	Physical disability	-	Germany	1283 children 6–16 y	Cross-sectional	Fair
Roberto et al. 2012 [30]	D, ND	Age	Sugar intake	Brazil	266 dental records 1–5 y	Cross-sectional	Poor – data obtained from dental records
Wyne et al. 2017 [31]	D	Age	-	Saudi Arabia	52 children	Cross-sectional	Poor – participant bias from survey
Gimenez-Prats et al. 2003 [32]	D	-	Sex	Spain	103 children 5–20 y in hospital	Cross-sectional	Fair
De Castilho et al. 2017 [33]	OC	Less bruxism and dental attrition	-	Brazil	171 records of female children 1–13 y	Cross-sectional	Fair
Subramaniam et al. 2014 [34]	OC	Low salivary antioxidant capacity Low salivary pH	-	India	34 non-institutionalised children 7–12 y vs. 33 matched healthy children	Case control	Good
Subramaniam et al. 2010 [35]	OC	Low salivary pH	-	India	100 non-institutionalised children 5–12 y vs. 100 matched healthy children	Case control	Good
Hegde et al. 2008 [36]	OC	-	Drooling	India	113 children 5–18 y attending special schools	Cross-sectional	Fair
Ruiz et al. 2018 [37]	OC	-	Salivary osmolality	Brazil	52 CP children in rehabilitation vs. 52 healthy children, 4–20 y	Case control	Good
Storhaug 1985 [38]	ND	Sugar intake	Sweetened medication	Norway	436 children 1–6 y attending health centre	Cross-sectional	Fair
Grzic et al. 2011 [39]	ND	-	Diet consistency	Croatia	43 institutionalised children 7–16 y vs. 43 matched healthy children	Case control	Good

**Table 2:** Quantification of significance and non-significance found for six risk factors and their respective sub-categories (N = 30) Socioeconomic factors (SE), Cerebral Palsy Subtype (CPS), Demographics (D), Condition of Oral Cavity (OC), Dental Habits (DH) and Nutrition and Diet (ND).

Risk Factor (Total Studies/N)	Sub-Category	No. of Studies Finding Significance	No. of Studies Finding No Significance
SE (8)	Low caregiver education	5	0
	Crowded household	2	0
	Low domestic income	1	5
CPS (14)	Low intellectual ability	3	0
	Subtype of spastic CP	3	4
	Neurological CP class (spastic)	2	0
	High GMFCS IV–V score	2	0
	Low general motor/physical ability	2	0
D (10)	Age	7	1
	Sex	1	3
OC (9)	Low oral motor control	2	0
	Salivary markers (antioxidants, pH)	2	0
	Salivary markers (flow, osmolality)	1	1 (osmolality)
	Drooling	1	1
	Less bruxism and dental attrition	1	0
	Biting reflex	1	0
DH (7)	Low tooth brushing frequency	4	1
	Dental visit frequency	2	1
	Dental habits of caregiver	2	0
ND (8)	Sugar intake	5	0
	Sweetened medication	0	1
	Food consistency (liquid)	1	3
	Snacking frequency	0	2

## Data Availability

All data and materials are available.

## Supplementary Materials

The following are available online at https://www.mdpi.com/article/10.3390/ijerph19138024/s1, Figure S1: PRISMA extension for scoping reviews (PRISMA-ScR) checklist pertaining to current study [53]; Figure S2: Search strategy used for PubMed database search including main search string, and filtered by year of publication, language, and population age.
